# *Hor*TILLUS—A Rich and Renewable Source of Induced Mutations for Forward/Reverse Genetics and Pre-breeding Programs in Barley (*Hordeum vulgare* L.)

**DOI:** 10.3389/fpls.2018.00216

**Published:** 2018-02-21

**Authors:** Miriam E. Szurman-Zubrzycka, Justyna Zbieszczyk, Marek Marzec, Janusz Jelonek, Beata Chmielewska, Marzena M. Kurowska, Milena Krok, Agata Daszkowska-Golec, Justyna Guzy-Wrobelska, Damian Gruszka, Monika Gajecka, Patrycja Gajewska, Magdalena Stolarek, Piotr Tylec, Paweł Sega, Sabina Lip, Monika Kudełko, Magdalena Lorek, Małgorzata Gorniak-Walas, Anna Malolepszy, Nina Podsiadlo, Katarzyna P. Szyrajew, Anete Keisa, Zodwa Mbambo, Elena Todorowska, Marek Gaj, Zygmunt Nita, Wanda Orlowska-Job, Miroslaw Maluszynski, Iwona Szarejko

**Affiliations:** ^1^Department of Genetics, Faculty of Biology and Environmental Protection, University of Silesia, Katowice, Poland; ^2^Department of Microbiology and Biotechnology, Faculty of Biology, University of Latvia, Riga, Latvia; ^3^Biosciences, Council for Scientific and Industrial Research, Pretoria, South Africa; ^4^Agrobioinstitute, Sofia, Bulgaria; ^5^Seed Company Plant Breeding Strzelce Ltd., Plant Breeding and Acclimatization Institute, Błonie, Poland

**Keywords:** TILLING, reverse genetics, mutation, barley, MNU, sodium azide

## Abstract

TILLING (Targeting Induced Local Lesions IN Genomes) is a strategy used for functional analysis of genes that combines the classical mutagenesis and a rapid, high-throughput identification of mutations within a gene of interest. TILLING has been initially developed as a discovery platform for functional genomics, but soon it has become a valuable tool in development of desired alleles for crop breeding, alternative to transgenic approach. Here we present the *Hor*TILLUS (***Hor**deum*—**TILL**ING—**U**niversity of **S**ilesia) population created for spring barley cultivar “Sebastian” after double-treatment of seeds with two chemical mutagens: sodium azide (NaN_3_) and N-methyl-N-nitrosourea (MNU). The population comprises more than 9,600 M_2_ plants from which DNA was isolated, seeds harvested, vacuum-packed, and deposited in seed bank. M_3_ progeny of 3,481 M_2_ individuals was grown in the field and phenotyped. The screening for mutations was performed for 32 genes related to different aspects of plant growth and development. For each gene fragment, 3,072–6,912 M_2_ plants were used for mutation identification using LI-COR sequencer. In total, 382 mutations were found in 182.2 Mb screened. The average mutation density in the *Hor*TILLUS, estimated as 1 mutation per 477 kb, is among the highest mutation densities reported for barley. The majority of mutations were G/C to A/T transitions, however about 8% transversions were also detected. Sixty-one percent of mutations found in coding regions were missense, 37.5% silent and 1.1% nonsense. In each gene, the missense mutations with a potential effect on protein function were identified. The *Hor*TILLUS platform is the largest of the TILLING populations reported for barley and best characterized. The population proved to be a useful tool, both in functional genomic studies and in forward selection of barley mutants with required phenotypic changes. We are constantly renewing the *Hor*TILLUS population, which makes it a permanent source of new mutations. We offer the usage of this valuable resource to the interested barley researchers on cooperative basis.

## Introduction

Mutants are essential for functional analysis of genes. There are many techniques, available today, of creating mutants that can be used to study gene function. Among these techniques there are standard chemical or physical mutagenic treatments causing mutations that are spread throughout the genome, as well as modern techniques of gene editing, such as CRISPR/Cas9-based system that can be used for creation of mutations within a specific target gene (Cong et al., 2013). The latter one, however, is still not available for many plant species, for which the protocols of transformation and/or *in vitro* regeneration are not available or are difficult to optimize for a wider range of genotypes. In the case of barley, such protocols are well established only for one variety “Golden Promise” (a Scottish cultivar developed in 1967; Lawrenson et al., 2015), while transformation efficiency of modern barley cultivars is still insufficient for a routine use. Therefore, the methods, such as TILLING (Targeting Induced Local Lesions IN Genomes), are still relevant for functional analysis of genes in many species, including barley.

TILLING is a strategy used for functional analysis of genes that combines the classical mutagenesis and a rapid, high-throughput identification of mutations within a gene of interest. TILLING has been initially developed as a discovery platform for functional genomics in *Arabidopsis thaliana* (McCallum et al., 2000). In a few years, besides Arabidopsis (Till et al., 2003), TILLING populations have been created for other model plants, e.g., *Lotus japonicus* (Perry et al., 2009) and *Medicago truncatula* (Le Signor et al., 2009), and model animals: fruit fly (Winkler et al., 2005), zebra fish (Wienholds et al., 2003), and rat (Smits et al., 2004). The main advantage of TILLING as a reverse genetics strategy is that it can be applied to any species, regardless of its genome size and ploidy level.

Soon TILLING has become a valuable tool in development of desired alleles for crop breeding (reviewed in Kurowska et al., 2011; Tadele, 2016). The utility of TILLING has been demonstrated in many crop species, including cereals: maize (Till et al., 2004), rice (Till et al., 2007; Suzuki et al., 2008), sorghum (Xin et al., 2008), durum (Uauy et al., 2009), bread (Slade et al., 2005; Uauy et al., 2009; Fitzgerald et al., 2010; Chen et al., 2012), and einkorn wheat (Rawat et al., 2012); legumes and oil crops: pea (Dalmais et al., 2008), common bean (Porch et al., 2009), soybean (Cooper et al., 2008), sunflower (Kumar et al., 2013), rapeseed (Gilchrist et al., 2013); vegetables: tomato (Gady et al., 2009; Minoia et al., 2010; Piron et al., 2010; Okabe et al., 2011), pumpkin (Vicente-Dólera et al., 2014), cucumber (Fraenkel et al., 2014), radish (Kohzuma et al., 2017); industrial species: cotton (Aslam et al., 2016), flax (Chantreau et al., 2013), tobacco (Reddy et al., 2012); and staple tropical crops, such as banana (Jankowicz-Cieslak et al., 2012).

Barley is among the most important cereal species grown worldwide. In the statistics showing the world grain production in 2016/2017, barley is at the fourth position in terms of the harvested acreage and quantity of produced grain, following corn, wheat, and rice (www.statista.com/statistics/263977/world-grain-production-by-type/). Barley grain is used mainly as animal fodder, as a source of malt in brewery industry and as a component of various health foods. Barley exhibits a high genetic adaptability to a wide range of environments and serves as a model cereal for studying mechanisms of adaptation to abiotic stresses (Dawson et al., 2015). The role of barley as an important crop species is reflected in development of molecular tools, among them TILLING, that assist its functional analysis and breeding. Over years several TILLING populations have been created for this crop (Caldwell et al., 2004; Talamè et al., 2008; Gottwald et al., 2009; Lababidi et al., 2009), and the release and assembling of barley genome sequence have facilitated the use of TILLING platforms for functional genomics studies (International Barley Genome Sequencing Consortium et al., 2012; Beier et al., 2017; Mascher et al., 2017).

In this paper we describe a new TILLING platform in barley created at the University of Silesia in Katowice, Poland. The population called *Hor*TILLUS (***Hor**deum vulgare*—**TILL**ING—**U**niversity of **S**ilesia)—was developed for spring barley variety “Sebastian” after double treatment with two chemical mutagens: sodium azide (NaN_3_) and N-Methyl-N-nitrosourea (MNU). It can serve as a platform for functional genomics and pre-breeding programs of barley. The *Hor*TILLUS population has proved its utility as a reverse genetics tool in many barley studies concerning e.g., brassinosteroid metabolism (Gruszka et al., 2016), DNA repair (Stolarek et al., 2015a,b), strigolactone signaling (Marzec et al., 2016), waterlogging tolerance (Mendiondo et al., 2016), or drought and ABA response (Daszkowska-Golec et al., 2017). This population is being constantly renewed to make it functional for many years. We present the *Hor*TILLUS platform as a renewable source of new mutations and mutants which we want to share within barley community in a cooperative manner.

## Materials and methods

### Material

Spring barley cultivar “Sebastian” (Supplementary Figure 1) was used as a parent variety to create the *Hor*TILLUS population. This cultivar was chosen because of its high yield potential, good malting quality, resistance to lodging and high resistance to stem rust (*Puccinia graminis*) and leaf rust (*Puccinia hordei*). It is also characterized by an average resistance to powdery mildew (*Blumeria graminis* f.sp. *hordei*), net blotch (*Pyrenophora teres*), and scald (*Rynchosporium secalis*).

### Mutagenesis

Seeds of “Sebastian” were mutagenized with the use of two mutagens: sodium azide (NaN_3_) and N-Methyl-N-nitrosourea (MNU), with inter-incubation germination period (iig) (Szarejko and Maluszynski, 1999; Szarejko et al., 2017). Two treatments were applied, both with the same dose of NaN_3_, but two different doses of MNU: a higher dose (1.5 mM NaN_3_/3 h–6 h iig – 0.75 mM MNU/3 h) and a lower dose (1.5 mM NaN_3_/3 h–6 h iig – 0.5 mM MNU/3 h).

### Creation of *Hor*TILLUS platform

**Figure 1:**
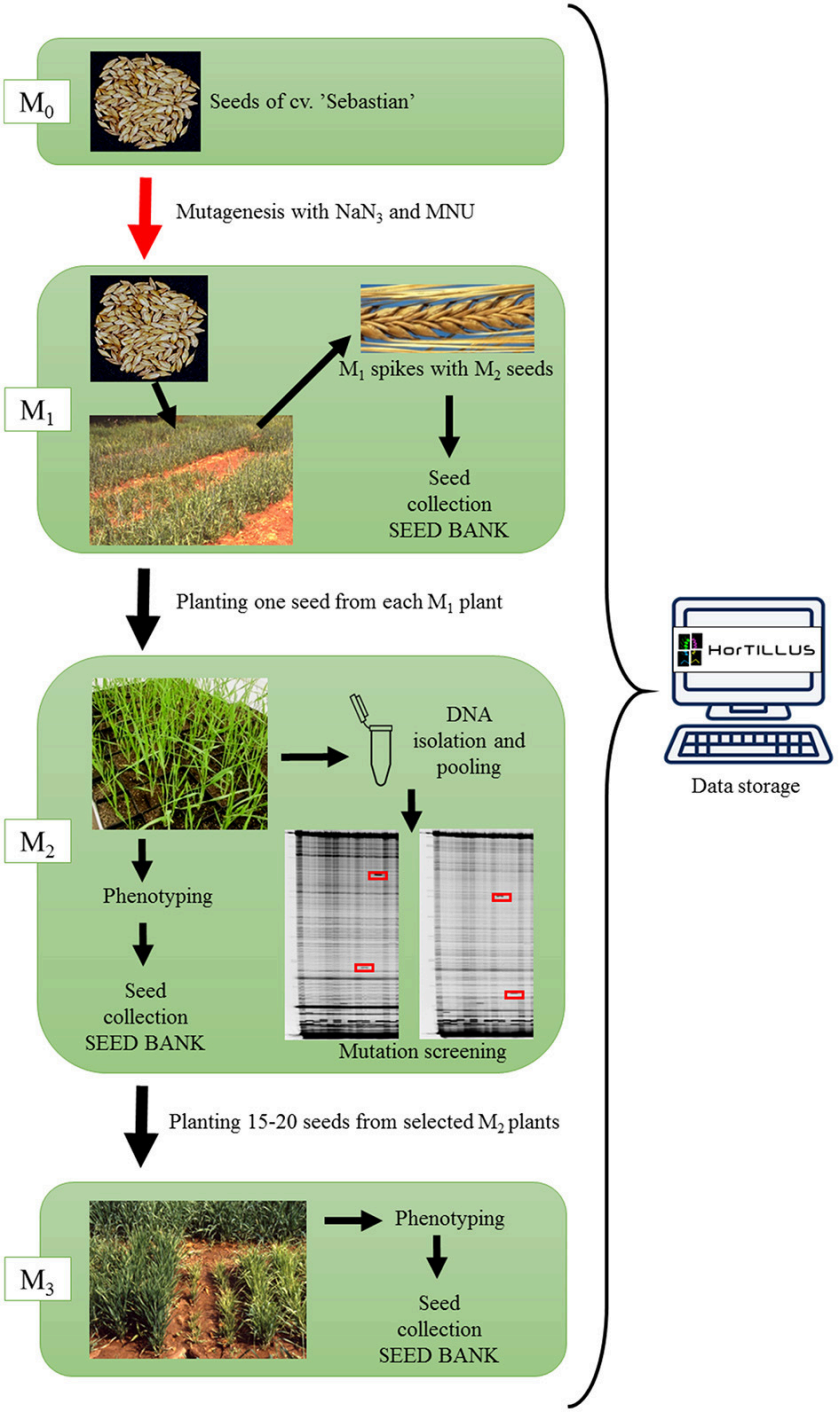
The scheme of *Hor*TILLUS population creation.

The scheme of creation of *Hor*TILLUS population is shown in Figure 1. The M_1_ population was grown at experimental field and harvested individually. All M_1_ spikes were stored but only one M_2_ plant was developed from each M_1_ plant in order to avoid repetitions of the same mutations in M_2_ progenies. After 2 weeks of seedlings hardening at 4°C, M_2_ plants were grown under controlled conditions in a greenhouse (photoperiod 16/8 h, light intensity 400 μM/m^2^/s and temperature 22/20°C during day and night, respectively) until harvest.

M_2_ generation of initial *Hor*TILLUS population has been created using almost 15,000 M_1_ plants developed after double treatment with sodium azide and MNU. Among those M_1_ plants, 4,716 were developed after treatment with a higher dose of MNU (1.5 mM NaN_3_/3 h–6 h iig – 0.75 mM MNU/3 h) and 10,116 after treatment with a lower dose of MNU (1.5 mM NaN_3_/3 h–6 h iig – 0.5 mM MNU/3 h). One seed from each M_1_ plant was planted and in total 13,181 M_2_ seeds germinated. DNA isolation and phenotypic analysis were performed on over 11,000 M_2_ plants.

All M_2_ plants were morphologically characterized at different developmental stages—from a seedling stage to maturity. During post-harvest analysis plants were measured and described for several characters, such as: number of tillers, fertility, number, and weight of seeds and any visible morphological changes. Mutants with visible changes were photographed using Fujifilm S9600 digital camera and the images were stored in a database.

Fifteen to twenty seeds from 3,481 individual M_2_ plants producing a high number of seeds were planted in the field belonging to Plant Breeding and Acclimatization Institute in Strzelce. M_3_ seeds from the same M_2_ plant were sown in two neighboring rows in plots and all M_3_ seedlings were carefully labeled. M_3_ progenies were phenotyped at different developmental stages, for different traits, such as presence of chlorophyll seedlings, shape and color of rosettes, morphology of leaves and spikes, time of flowering, height of plants, etc. M_3_ plants with morphological changes were also photographed. All data collected for individual plants and lines of M_3_ generation were stored in the database. M_4_ seeds were stored in a seed bank.

The proper storage of material in a seed bank is crucial for maintaining the TILLING population. Therefore, we vacuum-packed the seeds of individual plants from M_1_-M_4_ generations (using vacuum packer Tepro pp 5.4) and stored them at 4°C.

It should be stressed that after a few years of intensive usage, some seed samples of M_2_ plants were almost expended and/or the germination capacity of others decreased. To keep the *Hor*TILLUS population suitable for a long-term use we have decided to gradually renew our population by constantly growing new M_2_ plants and isolating DNA from them. These M_2_ plants derived from the same mutagenic treatments as those used for creation of the initial TILLING platform.

### DNA isolation and pooling

DNA was isolated from M_2_ *Hor*TILLUS plants individually using a modified micro-CTAB method (Doyle and Doyle, 1987) that ensures a large amount of high quality DNA. Leave samples (~3 cm^2^ segments) of 2–4-weeks-old plants were collected in silica gel in order to remove water from the tissue. Dried samples were ground in single eppendorf tubes with 3 mm glassballs (Sigma-Aldrich) using an electric mill (Retsch, MM200 and MM301). The exact protocol for DNA isolation from barley used for this study was described elsewhere (Szarejko et al., 2017). DNA was isolated from over 11,000 M_2_ plants.

DNA quantity and quality was measured using NanoDrop™ ND-1000 UV-Vis Spectrophotometer. Stocks were diluted to 100 ng/μl. DNA stocks were stored at −80°C, whereas the dilutions were stored in −20°C. The dilutions were used to prepare eight-fold pools in 96-well plates (Supplementary Figure 2). One well of the pool plate contains DNA from 8 different M_2_ plants, so one pool plate contains DNA from 768 M_2_ plants. Eight-fold pools served as a templates for PCR reactions.

### Gene and amplicon selection

Candidates for TILLING analysis were chosen based on literature data as genes potentially related to biological processes of our interest, such as brassinosteroid metabolism, response to abiotic stresses, root development or DNA repair (Table 1). GenBank (http://www.ncbi.nlm.nih.gov/genbank/) database was searched for sequences of candidates (mainly from Arabidopsis and rice). The selection of some genes was performed before the release of barley genome sequence (International Barley Genome Sequencing Consortium et al., 2012; Beier et al., 2017), therefore barley genomic sequences of candidates were unavailable. The homologous sequences of gene of interest from rice were retrieved in order to clone barley homologs. Rice mRNA or amino acid sequence were used as a query in barley Expressed Sequence Tags (EST) database (http://compbio.dfci.harvard.edu/tgi/). For the best of obtained hits primers were designed using i.e., Primer3 (http://bioinfo.ut.ee/primer3-0.4.0/) with the purpose of amplifying, sequencing and assembling a full coding sequences of barley homologs. After that, the barley coding sequence was aligned with the genomic sequence of the rice homolog using BLASTN algorithm or Splign (http://www.ncbi.nlm.nih.gov/sutils/splign/splign.cgi) what allowed to predict exon/intron putative borders in the barley gene and design primers for amplification of intron regions. Assembling of amplified sequences resulted in full barley genomic sequence. When the barley genome sequence became publicly available, it was possible to search directly for barley genes in databases, such as EnsemblPlants database (http://plants.ensembl.org/Hordeum_vulgare/Info/Index) or PLAZA 3.0 Monocots database (https://bioinformatics.psb.ugent.be/plaza/versions/plaza_v3_monocots/organism/view/Hordeum+vulgare).

**Table 1:** Candidate genes selected for TILLING analysis.

**Gene**	**Name**	**Acc.no of barley sequence**	**Protein function**
*Dhn5*	Dehydrin 5	AF181455	DHN5 (Dehydrin 5) belongs to group 2 LEA (Late Embryogenesis-Abundant) proteins that can be induced by salt and abscisic acid (ABA) (Brini et al., 2007)
*HVA1*	*Hordeum vulgare* Aleurone 1	X78205	HVA1 belongs to group 3 LEA (Late Embryogenesis-Abundant) proteins. Its expression can be induced by either treatment with abscisic acid (ABA) or by stress conditions such as drought, cold, heat, and salinity protein involved in cell membrane protection against dehydration (Straub et al., 1994)
*HvABI5*	ABA-Insensitive 5	HQ456390	ABI5 is a basic leucine zipper transcription factor that plays a key role in the regulation of seed germination and early seedling growth in the presence of ABA and abiotic stresses (Skubacz et al., 2016)
*HvAPY2*	Apyrase 2	HORVU4Hr1G087230	APY 2 is an enzyme that catalyzes the hydrolysis of extracellular ATP to yield AMP and inorganic phosphate (Yuo et al., 2009)
*HvBAK1*	BRI1-Associated Receptor Kinase1	HQ529501	BAK1 is a component of BR receptor involved in brassinosteroid perception (Gruszka et al., 2011)
*HvCBP20*	Cap Binding Protein 20	FJ548567	CBP20 (Cap-Binding Protein 20) is a small subunit of cap-binding complex (CBC) involved in RNA metabolism, splicing and miRNA biogenesis as well as in dynamic stress signaling pathway (Daszkowska-Golec, 2017; Daszkowska-Golec et al., 2017)
*HvCBP80*	Cap Binding Protein 80	nd	CBP80 (Cap-Binding Protein 80) is a large subunit of cap-binding complex (CBC) involved in RNA metabolism, splicing and miRNA biogenesis as well as in dynamic stress signaling pathway (Daszkowska-Golec, 2017)
*HvCENH3*	Centromeric Histone H3	JF419328	CENH3 is a centromere-specific histone 3 that is variant that is replacing H3 in centromeric nucleosomes and recruits many essential kinetochore proteins (Ravi et al., 2011)
*HvDMC1*	DNA Meiotic Recombinase 1	AF234170	DMC1 plays the central role in homologous recombination in meiosis by assembling at the sites of programmed DNA double strand breaks and carrying out a search for allelic DNA sequences located on homologous chromatids (Doutriaux et al., 1998)
*HvDREB1*	Dehydration Responsive Element Binding protein 1	DQ012941	DREB1 is a transcription factor from AP2/ERF (APETALA2/Ethylene-Responsive Factor) family, involved in response to drought, salt and ABA treatment (Guo et al., 2016)
*HvDRF1*	Dehydration Responsive Factor 1	AY223807	DRF1 is a transcription factor involved in abscisic acid (ABA)-mediated gene regulation. The expression of *HvDRF1* was upregulated in barley leaves and roots under drought, salt or ABA treatment, and in embryos during seed maturation (Xue and Loveridge, 2004)
*HvDWARF*	Dwarf	HQ619227	DWARF is an enzyme—brassinosteroid C6-oxidase, that takes part in brassinosteroid biosynthesis (Gruszka et al., 2011, 2016)
*HvERA1*	Enhanced Response to ABA 1	nd	ERA1 (Enhanced Response to ABA1) is a beta-subunit of farnesyltransferase that perform farnesylation of target proteins. It is a post-translational modification by which a farnesyl group is attached to the cysteine residue of the conserved CaaX motif on the carboxy-terminal of the target proteins. The role of ERA1 in ABA-dependent drought stress response was established in several plant species (Cutler et al., 1996; Wang et al., 2005; Manmathan et al., 2013; Ogata et al., 2017)
*HvEXPB1*	β-Expansin 1	AY351785	EXPB1 belongs to family of expansins that show cell-wall-loosening action. EXPB1 is involved in root hair formation (Kwaśniewski and Szarejko, 2006)
*HvGNA1*	Glucosamine-6-phosphate N-Acetyltransferase 1	HQ398329	GNA1 is an enzyme involved in *de novo* biosynthesis of UDP-N-acetylglucosamine—metabolite in glycosylation of proteins and lipids, that is required for proper root system development (Jiang et al., 2005)
*HvHPA1*	Histidinol Phosphate Aminotransferase 1	nd	HPA1 is an enzyme involved in synthesis of histidine and histidine homeostasis maintenance that is crucial for root system development (Mo et al., 2006)
*HvHTD1*	High Tillering and Dwarf 1	HORVU7Hr1G096970	HTD1 is involved in strigolactone biosynthesis. It is carotenoid isomerase that converts all-trans-β-carotene into 9′-cis-β-carotene (Alder et al., 2012)
*HvHTD2*	High Tillering and Dwarf 2	HORVU0Hr1G006450	HTD2 is involved in strigolactone biosynthesis. It is dioxygenase that cleaves 9-cis-β-carotene to produce 9-cis-β-apo-10′-carotenal (Alder et al., 2012)
*HvHTD3*	High Tillering and Dwarf 3	HORVU3Hr1G071170	HTD3 is involved in strigolactone biosynthesis. It is dioxygenase that cleaves 9-cis-β-carotene to produce 9-cis-β-apo-10′-carotenal (Alder et al., 2012)
*HvHTD4*	High Tillering and Dwarf 4	HORVU3Hr1G013470	HTD4 is involved in strigolactone biosynthesis. It is monooxygenase that catalyzes the oxidation of carlactone to produce ent-2′-epi-5-deoxystrigol (Zhang et al., 2014)
*HvHTD5*	High Tillering and Dwarf 5	HORVU7Hr1G003090	HTD5 is involved in strigolactone signaling. It is F-box protein that is a part of an SCF ubiquitin ligase protein complex (Ishikawa et al., 2005)
*HvHTD6*	High Tillering and Dwarf 6	AK368890	HTD6 is involved in strigolactone signaling. It is strigolactone receptor with hydrolase activity (Marzec et al., 2016)
*HvKu70*	ATP-dependent DNA helicase Ku70	JN116587	Ku70 and Ku80 make up the Ku heterodimer, which binds to DNA double-strand break ends and is required for the non-homologous end joining (NHEJ) pathway of DNA repair (Manova and Gruszka, 2015)
*HvKu80*	ATP-dependent DNA helicase Ku80	JN116588	Ku70 and Ku80 make up the Ku heterodimer, which binds to DNA double-strand break ends and is required for the non-homologous end joining (NHEJ) pathway of DNA repair (Stolarek et al., 2015b))
*HvLSD1*	Lesion Simulating Disease 1	nd	LSD1 is a regulator of hypersensitive response that is plant reaction to prevent spread of biotroph pathogens (Keisa et al., 2008)
*HvPARP3*	Poly(ADP-Ribose) Polymerase 3	HM366605	PARP3 enzyme belongs to family of proteins, which modify nuclear proteins by poly-ADP-ribosylation, that is required for DNA repair, regulation of apoptosis, and maintenance of genomic stability. PARP3 is taking part in cellular response to double-strand breaks (Stolarek et al., 2015a)
*HvPRT6*	Proteolysis 6	nd	PRT6 is N-recognin E3 ligase involved in N-end rule pathway, that controls plant responses to hypoxia (Mendiondo et al., 2016)
*HvRAA1*	Root Architecture Associated 1	JF703136	RAA1 is a regulatory factor of cell cycle that is involved in root system development (Ge et al., 2004)
*HvRTH3*	Roothair defective 3	JF421241	RTH3 is involved in cell expansion and cell wall biosynthesis (Hochholdinger et al., 2008)
*HvSNAC1*	Stress Responsive NAC1	JF796130	SNAC1 is a transcription factor from NAC (petunia **N**AM and Arabidopsis **A**TAF1, ATAF2 and **C**UC2) family, involved in response to drought, salt and ABA treatment (Hu et al., 2006)
*HvUVRD*	DNA helicase Ultra Violet Resistance D	ADJ94112	UVRD is a helicase involved in nucleotide excision repair (Gruszka et al., 2012a)
*HvWRKY38*	WRKY Transcription Factor 38	AY541586	WRKY38 is a transcription factor that is involved in stress response (Marè et al., 2004)

In order to analyze a gene with the use of TILLING strategy it is necessary to determine a fragment of a gene where a point mutation has the highest probability to occur and change a protein function. Each of the studied genes was analyzed using CODDLE software (Choosing codons to Optimize Discovery of Deleterious Lesions; http://blocks.fhcrc.org/proweb/coddle/). As an input, the genomic and coding sequences of barley genes were used. CODDLE performs a BLAST search of known proteins to find conserved regions. CODDLE is based on two algorithms—SIFT (Sorting Intolerant From Tolerant) and PSSM (Position Specific Scoring Matrix). Both these algorithms weigh the identity and frequency of the amino acids found at each position in the protein. CODDLE separately handles the prediction of changes that could truncate the protein and destabilize the RNA (nonsense changes and splice junction changes), and the prediction of missense changes which should alter function of the gene product (located in conserved amino acid blocks in the CDS). In an addition to CODDLE analysis, to verify the conserved regions within the protein of interest we performed alignments of homologous proteins using ClustalW (http://www.ebi.ac.uk/Tools/msa/clustalw2/). Because both agents used as mutagens for *Hor*TILLUS create mostly G/C to A/T transitions, also the GC content was analyzed within fragments chosen for analysis. Fragments for TILLING analysis were 700–1,200 bp in length and included a regions determined by the above described methods. Primers that allow amplification of chosen region(s) (Supplementary Table 1) were designed using i.e. Primer3 (http://primer3.ut.ee/).

### Mutation detection

The method of mutation detection used in described study consists of the following steps:

1. PCR on eight-fold DNA pools using differentially labeled primers (forward primer labeled with IRDye700, reverse primer labeled with IRDye800)
2. Heteroduplex formation by denaturation and slow renaturation of PCR products (heteroduplex with a single nucleotide mismatch appear only in pools that contain DNA from plant carrying a mutation within amplified fragment)
3. Enzymatic digestion using endonuclease (Cel-1) that specifically recognize and cuts heteroduplex in a mismatch position
4. Purification of products using ethanol
5. Electrophoresis in polyacrylamide gel in LI-COR Sequencer
6. Analysis of gel image and identification of positive pools containing DNA with potential mutation within amplified fragment—PCR products should be visible in both channels in LI-COR sequencer (700 and 800 nm). When heteroduplex is cut by Cel-1, one fragment contains only IRDye700 label, the other only IRDye800 label, that is why the bands indicating mutation are visible in different channels. The sum of length of this two corresponding bands should be equal to the length of PCR product
7. Identification of particular plants from positive pools carrying potential mutations by performing steps 1–6 on two-fold pools containing DNA of parental variety (non-mutated) mixed with DNA of each individual from positive pool separately
8. Sequencing of analyzed fragments from selected plants in order to confirm mutations and check their type and zygosity state.

IRDye700 and IRDye800 labeled primers as well as unlabeled ones were diluted to 20 μM and used to prepare primer “cocktail” (in a 3^*^: 2: 4^*^: 1 proportion for IRDye700 labeled forward, unlabeled forward, IRDye800 labeled reverse, unlabeled reverse primers, respectively). PCR reactions were performed in 20 μl volume and the concentration of PCR substrates, as well as the conditions of amplification were optimized for each pair of primers individually. After amplification, the heteroduplex formation was performed at 95°C per 3 min for initial denaturation, and then at 70°C per 20 s (x 70 cycles) −0.1°C per cycle for slow renaturation. After heteroduplex formation 10 μl of samples were treated with 20 μl of 0.1 × Celery Juice Extract (CJE) containing Cel I enzyme, which was kindly provided by B. Till from IAEA, Seibersdorf Laboratory, where it was isolated according to the protocol described previously (Till et al., 2006). The enzymatic cleavage was performed at 45°C for 15 min. The next step was purification of products with ethanol. Sixty microliters of 96% ethanol with 1% of sodium acetate were added to each sample and centrifuged at 4°C, 18,000 × g for 20 min in order to precipitate DNA fragments. Afterwards, the ethanol was removed and DNA pellets were washed in 30 μl of 70% ethanol and centrifuged in the same conditions as previously for 15 min. After drying the samples from the rest of ethanol at 80°C, 3 μl of STOP buffer, containing 5% of bromophenol blue-xylene, 40% of formamide, and 1% of EDTA, was added.

For the purpose of visualization of products, samples were denatured and the polyacrylamide electrophoresis was carried out in LI-COR sequencers (LI-COR 4300). Electrophoresis was performed in denaturating 6% polyacrylamide gel in 1xTBE (Tris—Boric Acid—EDTA) running buffer, at settings of 3,000 V, 30 mA, and 30 W.

### Creation of *Hor*TILLUS database

The phpMyAdmin program and the MySQL system were used for creating *Hor*TILLUS database. phpMyAdmin is one of the most popular tools with freeware license for administrating the databases. MySQL is a relational database management system (RDBMS) that runs as a server providing multi-user access to a number of databases.

*Hor*TILLUS database includes a detailed description of individual plants from M_2_ generation and their progenies. The data about plants from various generations are collected in individual tables due to differential analyses subjected to generations. Several tables describing inter alia: phenotype and molecular analysis were created for each of generation. All the tables are linked by dedicated key entries that enable the identification of a single M_2_ plant and all next-generation (M_3_, M_4_, M_5_) individuals derived from this plant. The information on the preceding generation (M_1_) is also provided. This resolution enables a flexible scanning of database contents and a simultaneous browsing of the data in different generations.

*Hor*TILLUS database includes description of changes in plant phenotypes and the information about mutations found in the analyzed genes (such as type of mutation, its precise position in TILLed fragment and zygosity state in M_2_ plant). Furthermore, there is information about fertility, number and weight of grains for each plant of M_1_, M_2_ generations and their progenies. The information about the number of grains used for phenotypic analyses and the number of grains left in the seed bank is also attached. All this options enable instant and simple supervision of seed bank status and, if necessary, multiplication of mutants which grains are running out.

The *Hor*TILLUS database contains detailed information about quality and concentration of DNA isolated from M_2_ plants and about its place of storage. Hence, it is possible to prepare new dilutions of DNA and re-stock existing DNA pools. One of the tables of the database includes the information about created DNA pools used in mutation searching. After finding a potential mutation in a pool, all eight M_2_ plants whose DNA were pooled in this single bulk can be easily identified.

*Hor*TILLUS database is supported by forms created in PHP and HTML languages. Therefore, it is possible to edit collected data and to enter new information using any Web browser. Created search scripts allow to ask a query about phenotype description and/or information about molecular analysis. Additionally, creation of database consisting of many individual tables allows their free modification and addition of extra tables for collecting additional information from any new studies.

## Results

### Status of initial *Hor*TILLUS platform

Among 13,181 germinated M_2_ plants, 10% were lethal. In half of the cases (5.1%) lethality was caused by chlorophyll defects that led to development of *albina, albinoviridis, striata, viridis*, or *xantha* seedlings. A higher dose of MNU (0.7 mM) led to the higher frequency of lethal seedlings (12.1%) than the 0.5 mM MNU mM used in a combination with the same dose of 1.5 mM NaN_3_ (9.1%). Eleven thousand one hundred and ninety-nine M_2_ plants have been grown till maturity, among them 87.4% were fertile and produced seeds (Table 2). All M_2_ plants were characterized for obvious morphological changes. Reduction of plant height was the most common alteration of phenotype—almost 8% of analyzed plants were dwarf (>50% shorter than the parental variety) or semi-dwarfs (50–75% height of the parental variety “Sebastian”). Other phenotypic changes observed in M_2_ plants include alterations in plant architecture, time of flowering, and changes in the development of rosette, leaves, spikes, and awns (Table 3), including alterations in shape, color, and sizes of these organs.

**Table 2:** Status of initial *Hor*TILLUS M_2_ generation.

**Categories**	**1.5 mM NaN_3_ −0.7 mM MNU**		**1.5 mM NaN_3_ −0.5 mM MNU**		**Total**	
	**No**.	**%**	**No**.	**%**	**No**.	**%**
Planted seeds	4,716	100	10,116	100	14,832	100
Developed seedlings	4,083	86.58	9,098	89.93	13,181	88.87
Seedlings declined	494	12.10^*^	827	9.09^*^	1,321	10.02^*^
Chlorophyll mutants	316	7.74^*^	345	3.79^*^	661	5.01^*^
- *albina*	189	4.63^*^	223	2.45^*^	412	3.12^*^
- *albinoviridis*	26	0.64^*^	41	0.45^*^	67	0.51^*^
- *striata*	2	0.05^*^	0	0^*^	2	0.02^*^
- *viridis*	38	0.93^*^	55	0.60^*^	93	0.71^*^
- *xantha*	61	1.49^*^	26	0.29^*^	87	0.66^*^
Plants grown till maturity	3,273	100	7,926	100	11,199	100
- Fertile/semi-fertile	2,766	84.5^**^	7,015	88.5^**^	9,781	87.4^**^
-Sterile	507	15.5^**^	911	11.5^**^	1,418	12.6^**^

* *The percentage was calculated in relation to the number of germinated seedlings*.

** *The percentage was calculated in relation to the number of plants grown till maturity*.

**Table 3:** Phenotypic changes observed in M_2_ generation.

**Categories**	**Subcategories**	**No. of M**_**2**_ **plants**	
		**1.5 mM NaN_3_ −0.7 mM MNU**	**1.5 mM NaN_3_ −0.5 mM MNU**
Height of plant	Dwarf	29	30
	Semi-dwarf	280	540
Plant architecture	Uniculm	9	23
	Short internodes	5	18
	Erectoid growth habit	5	1
	Branchy	7	5
Time of flowering	Earliness (*Praematurum*)	0	2
Rosette	Prostrate/semiprostrate	4	8
	Grass-like	7	5
Leaves	*Eceriferum*	3	5
	Light green color	6	12
	Short and curled	4	5
Spike	Deformed	9	1
	Dense	3	19
	*Ert-c*	0	1
	*Zeocrithon*	2	3
	*Laxatum*	10	24
	Six-rowed/Intermedium	4	6
	Short	28	40
	Long	10	3
	*Multiflorus*	1	0
Awn	Short (*Breviaristatum*)	5	6
	Semi-smooth	2	4
	Smooth	5	7
	Awnless	0	1
	Curly	4	9
No. of analyzed M_2_ plants in total		3,273	7,926
		11,199	

**Figure 2:**
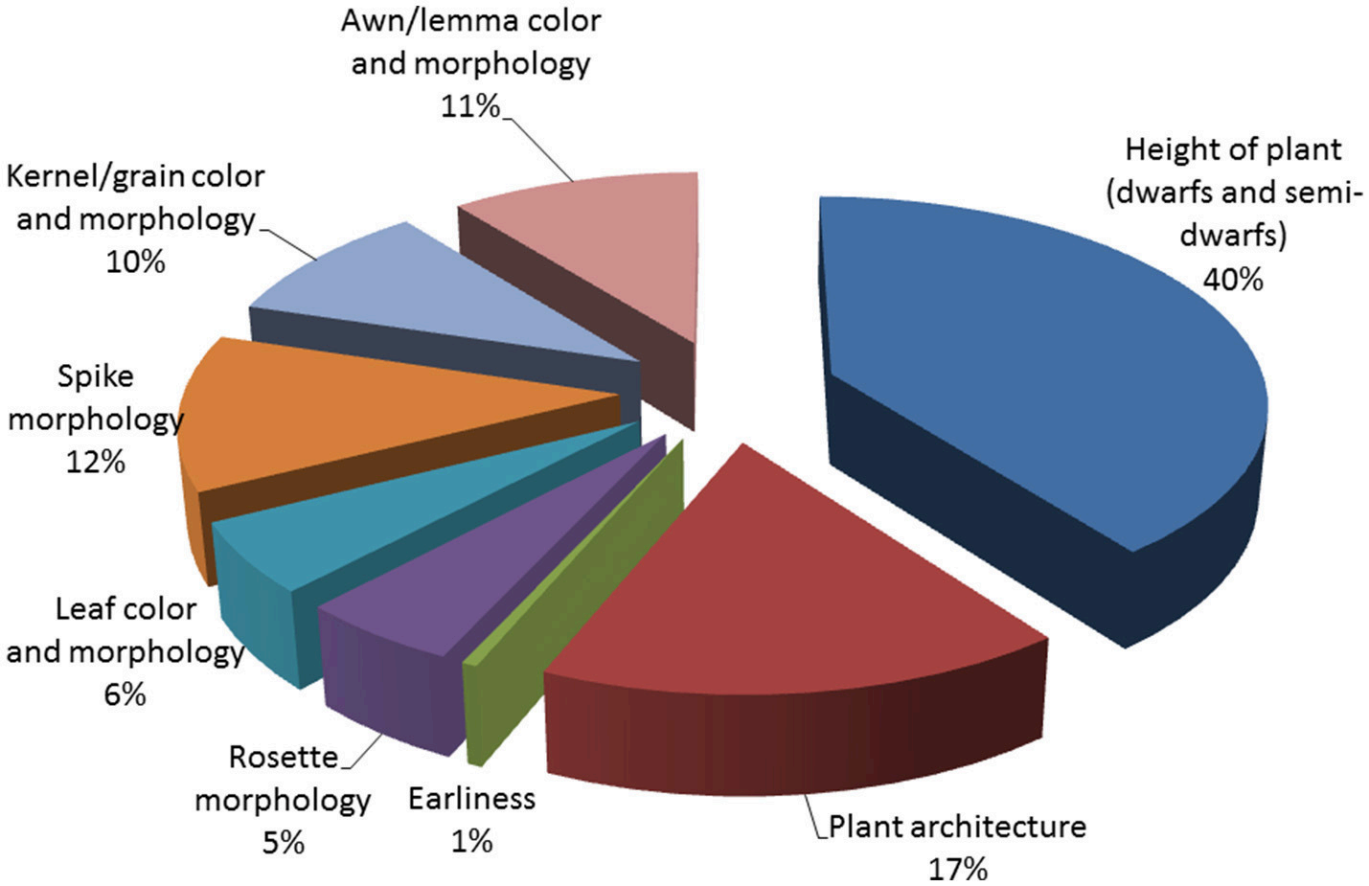
Types of morhpological alterations observed in M_3_ progenies (in %).

**Figure 3:**
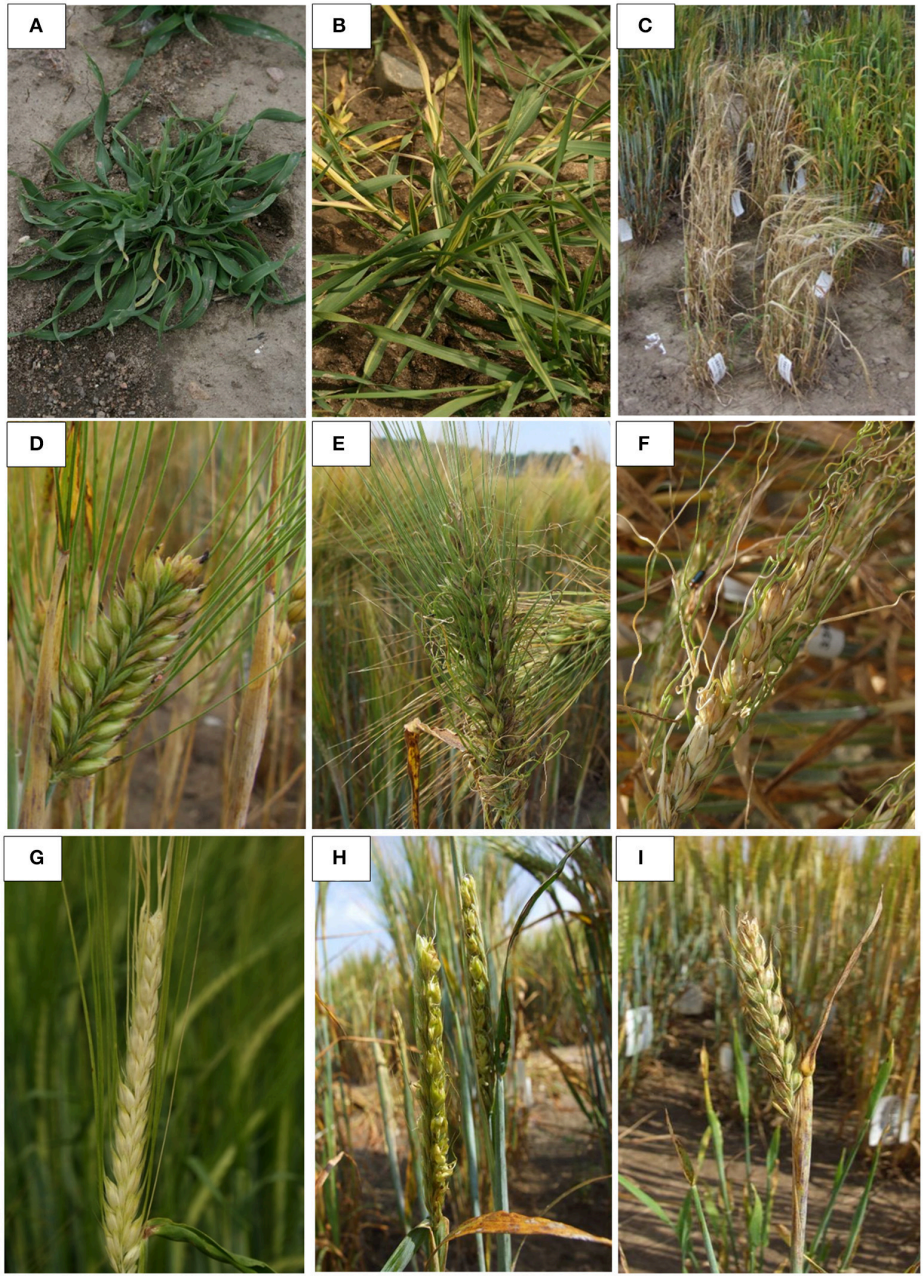
Examples of morphological changes observed in M_3_ *Hor*TILLUS plants: **(A)** curly prostrate rosette, **(B)** yellow stripped rosette, **(C)** earliness, **(D)** dense spike, **(E)** multiflorus, **(F)** curly awns, **(G)** white husk, **(H)** *Calcaroides*, **(I)** awnless.

We performed field observations of M_3_ progenies derived from 3,481 M_2_ plants that produced the highest amount of seeds, enough to provide a sample for M_3_ seed bank and M_3_ evaluation. Analysis of M_3_ families revealed that 30% of them carried morphological mutations. About one third of M_3_ families with morphological changes were homozygous for the observed phenotype but majority of M_3_ progenies segregated for one or more morphological characters (Table 4). Among these characters, alterations in plant height and growth habit were most often detected. We identified 138 homozygous M_3_ lines with dwarf or semi-dwarf plant height and 277 M_3_ families that segregated for this trait. Among changes of plant architecture, the *erectoid* growth type was most common (33 homozygous lines and 118 M_3_ families segregating for this character (Figure 2). Furthermore, forms exhibiting changes in time of flowering, color and morphology of leaves, color and shape of kernels and morphology of spikes and awns were observed in M_3_ generation (Figure 3). The frequency of M_3_ progenies with changes in these categories varied from ca.1% for early mutants to 15% for mutants with changes in plant growth type (Figure 2). To ensure a pedigree-based material for further forward mutant screens, we have harvested individually ca. 30,000 M_3_ plants and stored the vacuum-packed M_4_ seeds in refrigerator.

**Table 4:** Morphological changes analyzed in M_3_ progenies of 3,481 M_2_ plants.

**Category**	**Subcategory**	**No. of M**_**3**_ **progenies**	
		**Homozygous**	**Segregating**
Height of plant	Dwarf	13	14
	Semi-dwarf	125	263
Plant architecture	Uniculm	2	6
	Short internodes	0	0
	Erectoid growth habit	118	33
	Branchy	10	6
Time of flowering	Earliness (*Praematurum*)	2	3
Rosette	Prostrate/Semiprostrate	23	8
	Grass-like	8	15
Leaves	*Eceriferum*	5	5
	Light green color	36	7
	Short and curled	2	3
Spike	Dense	3	34
	*Ert-c*	0	13
	*Zeocrithon*	0	4
	*Laxatum*	3	16
	Six-rowed/Intermedium	0	5
	Short	1	30
	Long	0	7
	Lateral flower-less	0	4
	Small lateral flowers	0	5
	*Multiflorus*	0	1
Kernel/caryopsis	Necked	0	34
	Half necked	0	48
	White	0	1
	Black	0	1
	Purple	0	9
Grain	Unsymetrical	0	2
	Short and plump	1	4
Awn	Short (*Breviaristatum*)	0	8
	Semi-smooth	0	41
	Smooth	0	45
	Diverging	2	1
	Awn-less	0	0
	Hooded	0	2
	Curly	1	10
Lemma	Narrow	0	1
	Orange	1	0
No. of M_3_ families with morphological changes		356	689

### Mutation discovery

We have evaluated the usefulness of the *Hor*TILLUS population for mutation identification based on 32 gene TILLed. The genes which were chosen for this analysis were related to different aspects of plant growth and development, such as tolerance to abiotic stresses, growth regulators metabolism, or DNA repair (Table 1). Based on the CODDLE analysis, one to three gene fragments, for which there was the highest probability that mutation would affect an encoded protein function, were used for mutation detection. For each gene fragment, 3,072–6,912 M_2_ plants were used for mutation identification. In total, 182.16 Mb were screened in 40 gene fragments and 382 mutations were found (Table 5). Based on these results, we estimated the average mutation density in the *Hor*TILLUS population as 1 mutation per 477 kb. The mutation density varied from gene to gene—the highest (1/86 kb) was observed in *HvRAA1* and the lowest (1/7,066 kb) in one of the *HvKu70* fragments. Only in one fragment of *HvPARP3* gene we have not found any mutation. On average, we have identified 11.9 mutations per gene, with a range of 4–34 mutations. The density of mutations was higher in the *Hor*TILLUS subpopulation developed after treatment with a higher concentration of MNU (1 mutation per 414 kb) than in the subpopulation derived from treatment with a lower concentration of MNU, where 1 mutation was found per 608 kbp (Supplementary Table 2). This result is consistent with the frequency of chlorophyll mutants among M_2_ plants derived from these two treatments (7.7 and 3.8% after treatment with the higher and lower dose of mutagens, respectively).

**Table 5:** Mutation density in *Hor*TILLUS population based on 32 genes TILLed.

**Gene**	**No. of M_2_ plants analyzed**	**Length of amplicon (bp)**	**Amplicon GC content (%)**	**Amount of nucleotides scanned (bp)**	**No. of mutations detected**	**Mutation density (mut/kb)**
*Dhn5*	3,072	768	65.5	2,359,296	4	1/590
*HVA1*	3,072	700	65.1	2,150,400	16	1/134
*HvABI5*	6,144	1,072	62.9	6,586,368	28	1/235
*HvAPY2*	3,072	935	41.6	2,872,320	10	1/287
*HvBAK1*	3,072	778	39.2	2,390,016	6	1/398
*HvCBP20/*1	5,376	706	53.0	3,795,456	18	1/211
*HvCBP20/*2	5,376	730	39.4	3,924,480	11	1/357
*HvCBP20/*3	5,376	787	39.7	4,230,912	3	1/1,410
*HvCBP80*	3,072	691	39.5	2,122,752	12	1/177
*HvCENH3*	6,144	574	57.9	3,526,656	7	1/504
*HvDMC1*	5,376	811	41.6	4,359,936	6	1/727
*HvDREB1*	4,608	812	49.9	3,741,696	5	1/748
*HvDRF1*	3,072	724	51.2	2,224,128	16	1/139
*HvDWARF*	3,072	701	42.8	2,153,472	13	1/166
*HvERA1*	4,608	822	39.0	3,787,776	14	1/271
*HvEXPB1/1*	3,072	705	57.7	2,165,760	9	1/241
*HvEXPB1/2*	3,072	525	57.9	1,612,800	1	1/1,613
*HvGNA1*	5,376	552	67.7	2,967,552	12	1/247
*HvHPA1*	5,376	1,100	38.9	5,913,600	11	1/538
*HvHTD1*	6,912	971	60.9	6,711,552	16	1/419
*HvHTD2*	6,912	1,146	60.0	7,921,152	9	1/880
*HvHTD3*	6,144	1,126	40.1	6,918,144	9	1/769
*HvHTD4*	6,912	1,039	69.6	7,181,568	7	1/1,026
*HvHTD5*	6,912	1,084	75.5	7,492,608	9	1/833
*HvHTD6*	6,912	1,147	63.1	7,928,064	10	1/793
*HvKu70/1*	6,144	1,150	44.7	7,065,600	1	1/7,066
*HvKu70/2*	6,144	1,100	38.4	6,758,400	5	1/1,352
*HvKu70/3*	6,144	1,190	41.8	7,311,360	1	1/7,311
*HvKu80/1*	5,376	569	42.6	3,058,944	7	1/437
*HvKu80/2*	5,376	651	42.9	3,499,776	5	1/700
*HvLSD*	3,072	998	41.5	3,065,856	6	1/511
*HvPARP3/1*	5,376	527	44.2	2,833,152	1	1/2,833
*HvPARP3/2*	5,376	828	45.4	4,451,328	0	–
*HvPARP3/3*	5,376	805	46.3	4,327,680	10	1/433
*HvPRT6*	6,912	1,083	41.2	7,485,696	11	1/681
*HvRAA1*	5,376	545	58.8	2,929,920	34	1/86
*HvRTH3*	6,912	1,015	54.7	7,015,680	5	1/1,403
*HvSNAC1*	6,021	1,277	64.0	7,688,817	19	1/405
*HvUVRD*	6,144	877	39.5	5,388,288	9	1/599
*HvWRKY38*	3,072	730	63.5	2,242,560	6	1/374
Total				182,161,521	382	1/477

Mutagens used for creation of *Hor*TILLUS population caused mainly G/C to A/T transitions (88% of mutations; Table 6). Among them, 60% were G>A transitions and 40% C>T transition. Among nucleotide substitutions we have also found other types of transitions—T>C and A>G (4.5% of all mutations), as well as some transversions (C>A, G>T, A>C, C>G, T>G, and G>C). The transversions accounted for 7.5% of all mutations found in *Hor*TILLUS.

**Table 6:** Types of mutations found in *Hor*TILLUS population based on 32 genes TILLed.

**Gene**	**Nucleotide substitutions**									
	**Transitions**				**Transversions**					
	**G>A**	**C>T**	**T>C**	**A>G**	**C>A**	**G>T**	**A>C**	**C>G**	**T>G**	**G>C**
*Dhn5*	2	2	–	–	–	–	–	–	–	–
*HVA1*	12	3	1	–	–	–	–	–	–	–
*HvABI5*	17	8	–	–	1	–	–	1	1	–
*HvAPY2*	6	3	–	–	1	–	–	–	–	–
*HvBAK1*	6	–	–	–	–	–	–	–	–	–
*HvCBP20*	10	17	2	–	–	2	1	–	–	–
*HvCBP80*	6	5	–	–	–	1	–	–	–	–
*HvCENH3*	2	5	–	–	–	–	–	–	–	–
*HvDMC1*	3	3	–	–	–	–	–	–	–	–
*HvDREB1*	5	–	–	–	–	–	–	–	–	–
*HvDRF1*	10	3	2	–	1	–	–	–	–	–
*HvDWARF*	5	6	–	1	–	1	–	–	–	–
*HvERA5*	7	1	2	1	–	2	–	–	–	1
*HvEXPB1*	5	3	–	–	1	1	–	–	–	–
*HvGNA1*	7	5	–	–	–	–	–	–	–	–
*HvHPA1*	7	4	–	–	–	–	–	–	–	–
*HvHTD1*	6	7	1	2	–	–	–	–	–	–
*HvHTD2*	4	4	–	–	–	1	–	–	–	–
*HvHTD3*	5	3	–	–	1	–	–	–	–	–
*HvHTD4*	5	1	–	–	–	–	–	–	–	1
*HvHTD5*	3	3	1	1	1	–	–	–	–	–
*HvHTD6*	4	5	–	–	–	–	–	1	–	–
*HvKu70*	5	2	–	–	–	–	–	–	–	–
*HvKu80*	8	2	1	–	–	–	–	1	–	–
*HvLSD1*	3	3	–	–	–	–	–	–	–	–
*HvPARP3*	9	1	–	–	–	1	–	–	–	–
*HvPRT6*	7	4	–	–	–	–	–	–	–	–
*HvRAA1*	16	14	1	1	1	1	–	–	–	–
*HvRTH3*	2	3	–	–	–	–	–	–	–	–
*HvSNAC*	11	7	–	–	–	–	–	1	–	–
*HvUVRD*	2	4	–	–	–	3	–	–	–	–
*HvWRKY38*	2	3	–	–	1	–	–	–	–	–
Total	202	134	11	6	8	13	1	4	1	2
%	52.9	35.1	2.9	1.6	2.1	3.4	0.25	1.0	0.25	0.5

The available DNA sequences in which mutations were detected allowed for an investigation of whether the G/C to A/T transitions (the predominant type of mutations) are distributed randomly in barley genes or whether the local bias in the nucleotide composition surrounding the methylated guanine (O^6^-metG) exists. Therefore, we have examined 5′-NGN-3′ sequence context for G/C to A/T transitions in a subgroup of 12 TILLed genes (Table 7). Purines formed more than 90% of nucleotides observed at the−1 site, with predominant guanine (55.4%), whereas at position +1 there was no such a bias. The predominant local sequence context bias for G/C to A/T transitions observed in our population was 5′-RmetGN-3′.

**Table 7:** The nucleotides flanking the mutated guanine (O^6^-metG) in *Hor*TILLUS population, based on 12 genes TILLed.

	**Nucleotides surrounding meG in position**							
	−**1**				+**1**			
	**G**	**A**	**C**	**T**	**G**	**A**	**C**	**T**
Total	86	56	8	5	60	32	34	29
(%)	55.5	36.1	5.2	3.2	38.7	20.7	21.9	18.7

Among 382 identified mutations in *Hor*TILLUS population, 261 (68.3%) occurred in coding gene regions and 121 (31.7%) in noncoding regions, mostly introns (Supplementary Table 3). Converting these values to density of mutations gives 1 mutation per 444 kb and 1 mutation per 548 kb in the coding and noncoding sequences, respectively. Among mutations in the coding regions, 61.5% were missense, 37.5% were silent and 1% were nonsense (Table 8). Over 57% of all mutations found in the *Hor*TILLUS population were heterozygous, while 42% were in homozygous state in M_2_ generation.

**Table 8:** Spectrum of mutations identified in *Hor*TILLUS population, based on 32 genes TILLed.

**Gene**	**No. of changes**				**Mutation state**	
	**Coding region**			**Non-coding region**	**Homozygous**	**Heterozygous**
	**Missense**	**Silent**	**Nonsense**			
*Dhn5*	3	1	0	0	3	1
*HVA1*	8	6	0	2	8	8
*HvABI5*	17	9	0	2	8	20
*HvAPY2*	2	0	0	8	5	5
*HvBAK1*	5	0	0	1	3	3
*HvCBP20*	7	4	0	21	23	9
*HvCBP80*	9	2	0	1	6	6
*HvCENH3*	3	0	0	4	3	4
*HvDMC1*	3	0	0	3	3	3
*HvDREB1*	3	2	0	0	5	0
*HvDRF1*	12	4	0	0	9	7
*HvDWARF*	5	3	0	5	8	5
*HvERA5*	3	1	0	10	8	6
*HvEXPB1*	4	2	1	3	7	3
*HvGNA1*	6	5	0	1	6	6
*HvHPA1*	3	3	0	5	7	4
*HvHTD1*	5	6	0	5	7	9
*HvHTD2*	6	3	0	0	5	4
*HvHTD3*	3	1	0	5	7	2
*HvHTD4*	4	1	0	2	3	4
*HvHTD5*	5	3	0	1	3	6
*HvHTD6*	2	8	0	0	8	2
*HvKu70*	0	3	0	4	1	6
*HvKu80*	7	5	0	0	7	5
*HvLSD1*	1	1	0	4	0	6
*HvPARP3*	4	0	0	7	9	2
*HvPRT6*	5	3	0	3	5	6
*HvRAA1*	4	14	2	14	25	9
*HvRTH3*	3	2	0	0	4	1
*HvSNAC1*	12	4	0	3	11	8
*HvUVRD*	4	0	0	5	7	2
*HvWRKY38*	2	2	0	2	5	1
Total	160	98	3	121	219	163
% of mutation	68.3			31.7	57.3	42.7
	41.9	25.6	0.8			

## Discussion

The mutation density in *Hor*TILLUS population, calculated as 1 mutation per 477 kbp, is among the highest mutation densities reported for barley. In the other barley TILLING populations created till now after chemical mutagenesis, this value ranges from 1 mutation/2,500 kb to 1 mutation/374 kb (Caldwell et al., 2004; Talamè et al., 2008, 2009; Gottwald et al., 2009; Lababidi et al., 2009; Kurowska et al., 2012; Supplementary Table 4). It should be noted, however, that the mutation density estimated in these populations was based on the analysis of only 2–11 genes that included in total 4.5–52.3 Mb of barley sequences, while we have examined 32 genes and more than 182 Mb. The mutation density in three barley populations for which more than 2 genes were analyzed (*Hor*TILLUS, Barke, and TILLMore) does not differ significantly according to the non-parametric Anova test (Kruskal–Wallis; Supplementary Figure 3). The relatively high mutation density in *Hor*TILLUS confirms a high efficiency of mutagenesis using a combined treatment with sodium azide and MNU (Till et al., 2007).

The mutation density in *Hor*TILLUS varied between different gene fragments from 1/86 to 1/7,066 kbp. The reason for this phenomenon may be the local sequence context of nucleotides surrounding the methylated guanine. Guanine is the main target of methylating agents, such as EMS and MNU. Most of mutations induced by these agents arise from alkylation of guanine at the O^6^ position, which leads to the formation of O^6^-metG, the DNA lesion with the strongest mutagenic property (Kleibl, 2002). We have investigated the nucleotide composition in the relation to G/C to A/T transitions and we clearly demonstrated the strong specificity of the nucleotide surrounding the methylated guanine at the −1 position. In 90% of cases it was purine. A nonrandom distribution of mutated guanine was also shown in other studies (Richardson et al., 1987; Mironov et al., 1993; Kurowska et al., 2012), where the motif 5′-RmetGN-3′ was detected for 68–82% G/C to A/T transitions. It is thought that the base close to the lesion can strongly influence the mutagenic efficiency by the local repair deficiencies. Another reason for a wide range of mutation densities in different genes may be related to the differentiated robustness of the PCR reaction that depends on the specificity of primers or the efficiency of their association with template. Dyes attached to primers may change their 3D structure, what in some cases, may influence the efficiency of primer-DNA association. For this reason, when using labeled primers, the PCR efficiency, and thus mutation detection efficiency, could be lower for some fragments. To overcome this problem there is a possibility to use other mutation detection methods that do not require labeled primers. We have optimized the method of mutation identification with the use of Fragment Analyzer™ (Advanced Analytical Technologies) and we are applying it currently for TILLING analyses.

N-Methyl-N-nitrosourea (MNU), one of the mutagens used for development of *Hor*TILLUS platform, was applied also in several TILLING experiments, for example in *G. max* (Cooper et al., 2008) and *O. sativa* (Suzuki et al., 2008), where it caused only G/C to A/T transitions. Treatment of barley with sodium azide (the second mutagen used in our study) also leads mostly to this kind of transitions (Talamè et al., 2008). As expected, in *Hor*TILLUS population we found mainly G/C to A/T mutations (88%). Most of them (60%) were G to A transition, and 40% were C to T transitions. G to A transitions arise from O^6^ alkylation of guanine in the nontranscribed (sense) DNA strand, due to mispairing of O^6^-metG with thymine in the first replication cycle, which leads to its replacement by adenine in the subsequent replication round. Alkylation of guanine in the transcribed (antisense) strand results in the C to T transition. The higher proportion of G>A transitions observed in this study may indicate a slight bias in the repair of O^6^-metG lesions between DNA strands, with a more efficient repair of the transcribed strand. Similar bias was observed after MNU treatment in the *hprt* (hypoxanthine-guanine phosphoribosyltransferase) gene in rat fibroblasts (Jansen et al., 1994). In *Hor*TILLUS population we also found other types of transitions—T to C and A to G. The T/A to C/G mutations may arise from methylation of O^4^ position of thymine which appears with a much lower frequency compared to O^6−^meG (Kleibl, 2002). In the first replication cycle, O^4^-metT mispairs with guanine and in the subsequent cycle it leads to the T/A to C/G transition. In our TILLING population this type of nucleotide substitutions occurred with a low frequency (4.5%). Besides transitions we found also some transversions (7.5% of all mutations). Sodium azide, used as one of the mutagens in our experiments, is known to be highly mutagenic in barley (Nilan et al., 1973) and other crop species, whereas it is marginally mutagenic in *A. thaliana* and mammals (Gruszka et al., 2012b). It leads mainly to transitions, but also to transversions (Olsen et al., 1993). Thus, the transversions observed in *Hor*TILLUS population might be caused by mutagenic activity of NaN_3_, although MNU is known to induce a relatively high level of transversions as well (Kurowska et al., 2012).

Interestingly, the comparison of alterations in coding and noncoding regions of TILLed DNA fragments showed a slightly higher mutation density in the coding sequences (1/444 vs. 1/548 kb, respectively). The noncoding regions represented introns, and in some cases the 5′ and 3′UTR. The slightly higher mutation density in the coding regions shows a lack of preference in mutagenic action and/or repair system in relation to the type of sequences. The most useful mutations in terms of functional genetics are changes that affect protein function, mainly missense, nonsense and splice junction mutations. Based on 32 genes TILLed, the majority of mutations occurring in coding sequences in *Hor*TILLUS population were missense (61%). We have also found three independent nonsense mutations leading to the premature STOP codon, while 98 mutations (37% of changes occurring in coding regions) were silent. These results are in agreement with the spectrum of mutations observed after EMS or sodium azide treatment in barley (Caldwell et al., 2004; Talamè et al., 2008; Gottwald et al., 2009) and clearly show that *Hor*TILLUS population is an useful reverse genetics tool.

Most of the mutations found in M_2_ plants were in heterozygous state (57%). The ratio of homozygous to heterozygous mutations in M_2_ generation should be 1:2 (Till et al., 2006). The lower level of heterozygotes among analyzed plants with mutations could be connected with a method of mutation detection applied in the study. When using the described LI-COR method of mutation identification, signals of heterozygous mutations are by half weaker than the signals of homozygous ones in the eight-fold DNA pools. Therefore, some of heterozygous mutations could be missed during analysis of LI-COR gel images.

One of the problems often raised in regards to application of TILLING platforms as a reverse genetics tool is a high density of background mutations that may affect the plant phenotype. Based on the analysis of *Hor*TILLUS population presented above, we estimate the average mutation density as ca. 1 mutation per 500 kb. This density gives about 10,000 mutations (nucleotide substitutions) in barley genome whose size is around 5 Gb. Taking into consideration only the coding part of barley genome, which size is estimated at 65.3 Mb (1.4%; Mascher et al., 2017), the number of mutations in coding sequences may account for ca. 130 mutations. Therefore, for functional genomics studies we recommend to overcome the problem of off target mutations by backcrossing the identified mutant to the parent variety. After two backcrosses this number will be reduced by 75%, giving about 32 background mutations left. As our analysis showed that about 40% of identified mutations are silent, this further reduces the number of off target mutations with potential effect, to about 19 in barley genome. In addition, when we work with TILLING as a reverse genetics strategy, we usually know the biological process in which a candidate gene is involved. Therefore, we analyze the identified mutant according to the phenotypic characters related to this process. To confirm that the identified mutation is really responsible for a phenotypic change in the mutant, we always perform a co-segregation analysis in F_2_ progeny of mutant x parent variety, and/or we evaluate a set of allelic mutants with different changes in the gene. We are aware that TILLING approach may be time and labor consuming but it is the only strategy available for plants with large genomes and/or poorly studied to produce mutants in genes of interest. The new strategy of genome editing (CRISPR-Cas9), which was reported also for barley (Lawrenson et al., 2015), is up to now restricted to one barley variety “Golden Promise,” due to difficulties in transformation of other barley cultivars. Additionally, TILLING is not a GMO strategy, which may be considered as an advantage in some countries.

*Hor*TILLUS is one of few TILLING platforms created for barley. In Supplementary Table 4 we have summarized the characteristics of all barley TILLING populations developed till now. The *Hor*TILLUS platform is the largest of those populations and is well characterized. It has been evaluated in terms of induced mutations in a wide range of genes located across barley genome and characterized for phenotypic changes in a large M_3_ population. The *Hor*TILLUS platform has already proven its usefulness in functional genomics studies. The platform has been utilized to reveal function of genes involved in brassinosteroid and strigolactone metabolism (Gruszka et al., 2016; Marzec et al., 2016), DNA repair (Stolarek et al., 2015a,b), tolerance to abiotic stresses (Mendiondo et al., 2016; Daszkowska-Golec et al., 2017). In addition, the individual collection of ca. 30,000 M_3_ progenies gives a unique basis for forward screening for mutants of interest. We would like to emphasize that our *Hor*TILLUS population is available to the barley community on a cooperative basis.

## Conclusion

We have created *Hor*TILLUS—the TILLING platform for barley with an average mutation density 1/477 kb, calculated on the basis of 32 gene TILLed. This platform proved to be a useful tool, both in functional genomic studies and in forward selection of barley mutants with required phenotypic changes, including agronomically important traits. A great advantage of the *Hor*TILLUS platform, compared to the other TILLING populations in barley, is that we are constantly renewing it by replacing the M_2_ plants, whose DNA and/or seeds are depleted, with the new ones. This makes *Hor*TILLUS a permanent source of mutations that can be used for many years. We offer the usage of this valuable resource to the interested barley researchers on a cooperative basis.

## Author contributions

IS and MMal: Conceived and designed the study; JZ: Performed mutagenesis; JZ, JJ: Handled M_1_ and M_2_ generations; JJ, MS-Z: Isolated DNA and prepared pools; JZ, MS-Z, BC, MMar: Phenotyped M_2_ plants; MS-Z, MMar, JJ, BC, MKur, MKr, AD-G, JG-W, DG, MGaje, PG, MS, PT, PS, SL, MKud, ML, MG-W, AM, NP, KS, AK, ZM, ET: Performed mutation screening of different fragments; JZ, MGaj, MS-Z, MMar, DG, WO-J, ZN: Handled and phenotyped M_3_ population; JZ: Maintained seed bank; MMar: Created the database; MS-Z and IS: Wrote the manuscript.

### Conflict of interest statement

The authors declare that the research was conducted in the absence of any commercial or financial relationships that could be construed as a potential conflict of interest.
