# Supplementary material for: HorTILLUS—A Rich and Renewable Source of Induced Mutations for Forward/Reverse Genetics and Pre-breeding Programs in Barley (Hordeum vulgare L.)
Source: Front Plant Sci. 2018 Feb 21;9:216. doi: 10.3389/fpls.2018.00216 (PMC5826354; doi:10.3389/fpls.2018.00216)
Supplement: Supplementary file 1 [file Table1.DOCX]

Supplementary Table 1. Primers used for TILLING analysis.

| **Gene** | **Primer Forward** | **Primer Reverse** |
| --- | --- | --- |
| ***Dhn5*** | ACAAGCAGGCCGCTGACAC | TCGCTCCGGTGTGTTCATGT |
| ***HVA1*** | GCAAGTCGATCCATTCCAAGTGA | GCCTGCTGGAGGACGCTG |
| ***HvABI5*** | AGGCAGGGGTCCATCTATTC | CCTCGTTCAGATCCTTGAGC |
| ***HvAPY2*** | TTTTATACCAATGGAATGTGCAG | AGTTAGCGGTCCACAGAGCTT |
| ***HvBAK1*** | TGGCTGTACACAGAAACTTATTG | CCAAGCACTTCTTTTTCCATT |
| ***HvCBP20/1*** | GACAAGCGCCGTCGACCT | GCTACGCCACATTTACAGTGGGA |
| ***HvCBP20/2*** | CCAGGGAAGATGCTGAAGATGC | GGAACGGAGGGAGTATTACGGA |
| ***HvCBP20/3*** | ATAAAATTGCCAAGCCGCTAT | TGCTGCCATATTCAAATGCCC |
| ***HvCBP80*** | ATTGAAGATGCTGCCGAGTT | GGCATGCCAATCAGACAGA |
| ***HvCENH3*** | CTCCCTCCTTCCTCCTAACC | CTAAGGGCAAAGGACAGCAG |
| ***HvDMC1*** | AGGTCAGGGAAGACCCAGTT | TGCTGCAGTCACAGAACACA |
| ***HvDREB1*** | TGGTACCCAACCCAAGTGAT | TGCTCCTCATGGGTACTTCC |
| ***HvDRF1*** | ATGGCCCTAATTCCGTCTCT | ACAGTCACCGGGTCAACTTC |
| ***HvDWARF*** | CTGATGATGGTTTCACCTTTGA | CAGGCGTGGATAAACAAAAGA |
| ***HvERA1*** | GTTACCTCATCTCGCTACATCG | AATCAAGCTAGGCAAATCGAG |
| ***HvEXPB1/1*** | GGTGAGCCTCGCGTACATTC | CGCCTTCAGCAAGCACACTC |
| ***HvEXPB1/2*** | CGTGCGTGACACTGGTTAGT | AAGCCTGAGCGATAATTGGA |
| ***HvGNA1*** | GACCCAGATGGCATCCAC | ATGCGACGAGACAAAGGAAT |
| ***HvHPA1*** | CCCTTATGTGTACCCTGATCCTGA | GGTCCAACAGACGTATTAGCCAAG |
| ***HvHTD1*** | GGAAACATGGCTACCAAGGA | GCTCAGAGCAATGGACCAATA |
| ***HvHTD2*** | GCATGGATCTGCATGCTCTGTT | GAGCGAGTAAGACGTTCACGAAAC |
| ***HvHTD3*** | GGAAACATGGCTACCAAGGA | GCTCAGAGCAATGGACCAATA |
| ***HvHTD4*** | AGCTGCTGCAGATCCTGAAC | TTTGCGTGTCCCTCTCTACC |
| ***HvHTD5*** | AGGGCCTAGAGACGAGAGGT | TTGTACCGTGTACGCACGTC |
| ***HvHTD6*** | AGGGCCTAGAGACGAGAGGT | TTGTACCGTGTACGCACGTC |
| ***HvKu70/1*** | ATGGACCTGGACCCCGAGGGC | ACACGGTGCAAAGACAAACAG |
| ***HvKu70/2*** | TTATAATCTCTGTTTTCACCTGTGC | GAGTTGAAAGCTCCCACTCG |
| ***HvKu70/3*** | CAAGATGAGCCACCTGGAAT | CCTCTTCTTTGAGGCACCAC |
| ***HvKu80/1*** | GTTCTGGATGCTATTGTTGT | ATAGGCCCACACCTGTTCAC |
| ***HvKu80/2*** | AAAGCAGCCAGATGCAAATC | TTGCTACACGCCTACACCTG |
| ***HvLSD1*** | CGCAATGCAACTACTGTCAGATGCTCA | CGAATGCAGACTGATTGGAAGGTGGTA |
| ***HvPARP3/1*** | TGAAGCAGCAAGAAGGGAAG | AAGGGTAATGTGCAGGCAAG |
| ***HvPARP3/2*** | GGAGCGTGGAACTCCTGTAG | CACGTCGAAACCAACATCCT |
| ***HvPARP3/3*** | GGATGATCCGCTGTCTGAAT | TTGTGTCCGAAACACAAGGA |
| ***HvPRT6*** | TGTCATGATCGATATTTGTTTTCC | TCGCTTAGTAGCATCCAAAAGA |
| ***HvRAA1*** | GTCGACGACTTGCATCATCTATCG | CACCCCGATCACTAACAACAACAA |
| ***HvRTH3*** | AATCGGCCTTCCAAATGCAG | GCGTTGATGGAACCACCTGA |
| ***HvSNAC1*** | CTCCTCTCACTCCCCAACAA | GTCATCCATTCCGCTTCTGT |
| ***HvUVRD*** | CAGCTGCTTTGATGAGCTGT | CCACAAGCATGACCAAATGT |
| ***HvWRKY38*** | ACCACCAGAGCCAATACCAG | TCTGCACCTTCTTCTTGACG |
