# Supplementary material for: HorTILLUS—A Rich and Renewable Source of Induced Mutations for Forward/Reverse Genetics and Pre-breeding Programs in Barley (Hordeum vulgare L.)
Source: Front Plant Sci. 2018 Feb 21;9:216. doi: 10.3389/fpls.2018.00216 (PMC5826354; doi:10.3389/fpls.2018.00216)
Supplement: Supplementary file 2 [file Table2.DOCX]

Supplementary Table 2. Comparison of mutation density in *Hor*TILLUS M_2_ plants after two different mutagenic treatments based on 25 genes that were screened in both sub-populations.

| **Gene** | **1.5 mM NaN_3_/3h - 6h iig - 0.75 mM MNU/3h** | | | | **1.5 mM NaN_3_/3h - 6h iig - 0.5 mM MNU/3h** | | | |
| --- | --- | --- | --- | --- | --- | --- | --- | --- |
|  | **No. of M_2_ plants analyzed** | **Nucleotides scanned** | **No. of mutations** | **Mutation density** | **No. of M_2_ plants analyzed** | **Nucleotides scanned** | **No. of mutations** | **Mutation density** |
| ***HvABI5*** | 3,272 | 3,507,584 | 18 | 1/195 | 2,872 | 3,078,784 | 10 | 1/308 |
| ***HvCBP20*** | 3,272 | 7,273,656 | 20 | 1/364 | 2,104 | 4,677,192 | 12 | 1/425 |
| ***HvCBP80*** | 2,304 | 1,592,064 | 9 | 1/177 | 768 | 530,688 | 3 | 1/177 |
| ***HvCENH3*** | 3,272 | 1,878,128 | 3 | 1/626 | 2,872 | 1,648,528 | 4 | 1/412 |
| ***HvDMC1*** | 3,272 | 2,653,592 | 6 | 1/442 | 2,104 | 1,706,344 | 0 | - |
| ***HvDREB1*** | 2,504 | 2,033,248 | 2 | 1/1017 | 2,104 | 1,708,448 | 3 | 1/569 |
| ***HvERA1*** | 3,272 | 2,689,584 | 5 | 1/538 | 1,336 | 1,098,192 | 9 | 1/122 |
| ***HvGNA1*** | 3,272 | 1,806,144 | 9 | 1/201 | 2,104 | 1,161,408 | 3 | 1/387 |
| ***HvHPA1*** | 3,272 | 3,599,200 | 6 | 1/600 | 2,104 | 2,314,400 | 5 | 1/463 |
| ***HvHTD1*** | 3,272 | 3,546,848 | 8 | 1/443 | 3,640 | 3,945,760 | 8 | 1/493 |
| ***HvHTD2*** | 3,272 | 3,749,712 | 6 | 1/625 | 3,640 | 4,171,440 | 3 | 1/1390 |
| ***HvHTD3*** | 3,272 | 3,684,272 | 7 | 1/526 | 2,872 | 3,233,872 | 2 | 1/1617 |
| ***HvHTD4*** | 3,272 | 3,399,608 | 3 | 1/1133 | 3,640 | 3,781,960 | 4 | 1/945 |
| ***HvHTD5*** | 3,272 | 3,546,848 | 2 | 1/1773 | 3,640 | 3,945,760 | 7 | 1/564 |
| ***HvHTD6*** | 3,272 | 3,752,984 | 2 | 1/1876 | 3,640 | 4,175,080 | 8 | 1/522 |
| ***HvKu70*** | 3,272 | 11,262,224 | 5 | 1/2252 | 2,872 | 9,885,424 | 2 | 1/4943 |
| ***HvKu80*** | 3,272 | 3,664,640 | 9 | 1/407 | 2,104 | 2,356,480 | 3 | 1/785 |
| ***HvLSD*** | 2,483 | 2,470,585 | 6 | 1/412 | 589 | 586,055 | 0 | - |
| ***HvPARP3*** | 3,272 | 7,064,248 | 6 | 1/1177 | 2,104 | 4,542,536 | 5 | 1/909 |
| ***HvPRT6*** | 3,272 | 3,543,576 | 8 | 1/443 | 3,640 | 3,942,120 | 3 | 1/1314 |
| ***HvRAA1*** | 3,272 | 1,783,240 | 21 | 1/85 | 2,104 | 1,146,680 | 13 | 1/88 |
| ***HvRTH3*** | 3,272 | 3,321,080 | 3 | 1/1107 | 3,640 | 3,694,600 | 2 | 1/1847 |
| ***HvSNAC1*** | 3,272 | 4,178,344 | 9 | 1/464 | 2,872 | 3,667,544 | 10 | 1/367 |
| ***HvUVRD*** | 3,272 | 2,617,600 | 6 | 1/436 | 2,872 | 2,297,600 | 3 | 1/766 |
| ***HvWRKY38*** | 968 | 706,640 | 5 | 1/141 | 2,104 | 1,535,920 | 1 | 1/1536 |
| **Total** |  | **107,263,057** | **259** | **1/414** |  | **74832815** | **123** | **1/608** |
