# Supplementary material for: HorTILLUS—A Rich and Renewable Source of Induced Mutations for Forward/Reverse Genetics and Pre-breeding Programs in Barley (Hordeum vulgare L.)
Source: Front Plant Sci. 2018 Feb 21;9:216. doi: 10.3389/fpls.2018.00216 (PMC5826354; doi:10.3389/fpls.2018.00216)
Supplement: Supplementary file 3 [file Table3.DOCX]

Supplementary Table 3. Density of mutations in coding and noncoding regions based on 32 genes TILLed.

| **Gene** | **No. of M_2_ plants analysed** | **Coding sequence** | | | | **Noncoding sequence** | | | |
| --- | --- | --- | --- | --- | --- | --- | --- | --- | --- |
|  |  | **Sequence length analysed** | **Nucleotides scanned** | **No. of mutations** | **Mutation density** | **Sequence length analysed** | **Nucleotides scanned** | **No. of mutations** | **Mutation density** |
| ***Dhn5*** | 3,072 | 768 | 2,359,296 | 4 | 1/590 | 0 | 0 | 0 | - |
| ***HVA1*** | 3,072 | 494 | 1,517,568 | 14 | 1/108 | 206 | 632,832 | 2 | 1/316 |
| ***HvABI5*** | 6,144 | 867 | 5,326,848 | 26 | 1/205 | 205 | 1,259,520 | 2 | 1/630 |
| ***HvAPY2*** | 3,072 | 542 | 1,665,024 | 2 | 1/833 | 392 | 1,204,224 | 8 | 1/151 |
| ***HvBAK1*** | 3,072 | 508 | 1,560,576 | 5 | 1/312 | 270 | 829,440 | 1 | 1/829 |
| ***HvCBP20*** | 5,376 | 715 | 3,843,840 | 11 | 1/349 | 1508 | 8,107,008 | 21 | 1/386 |
| ***HvCBP80*** | 3,072 | 498 | 1,529,856 | 11 | 1/139 | 193 | 592,896 | 1 | 1/593 |
| ***HvCENH3*** | 6,144 | 172 | 1,056,768 | 3 | 1/352 | 402 | 2,469,888 | 4 | 1/617 |
| ***HvDMC1*** | 5,376 | 338 | 1,817,088 | 3 | 1/606 | 473 | 2,542,848 | 3 | 1/848 |
| ***HvDREB1*** | 4,608 | 601 | 2,769,408 | 5 | 1/554 | 211 | 972,288 | 0 |  |
| ***HvDRF1*** | 3,072 | 724 | 2,224,128 | 16 | 1/139 | 0 | 0 | 0 |  |
| ***HvDWARF*** | 3,072 | 399 | 1,225,728 | 8 | 1/153 | 302 | 927,744 | 5 | 1/186 |
| ***HvERA5*** | 4,608 | 385 | 1,774,080 | 4 | 1/444 | 457 | 2,105,856 | 10 | 1/211 |
| ***HvEXPB1*** | 3,072 | 878 | 2,697,216 | 7 | 1/385 | 352 | 1,081,344 | 3 | 1/360 |
| ***HvGNA1*** | 5,376 | 492 | 2,644,992 | 11 | 1/240 | 60 | 322,560 | 1 | 1/323 |
| ***HvHPA1*** | 5,376 | 634 | 3,408,384 | 6 | 1/568 | 466 | 2,505,216 | 5 | 1/501 |
| ***HvHTD1*** | 6,912 | 628 | 4,340,736 | 11 | 1/395 | 343 | 2,370,816 | 5 | 1/474 |
| ***HvHTD2*** | 6,912 | 938 | 6,483,456 | 9 | 1/720 | 208 | 1,437,696 | 0 |  |
| ***HvHTD3*** | 6,144 | 842 | 5,173,248 | 4 | 1/1293 | 284 | 1,744,896 | 5 | 1/349 |
| ***HvHTD4*** | 6,912 | 794 | 5,488,128 | 5 | 1/1098 | 245 | 1,693,440 | 2 | 1/847 |
| ***HvHTD5*** | 6,912 | 976 | 6,746,112 | 8 | 1/843 | 108 | 746,496 | 1 | 1/746 |
| ***HvHTD6*** | 6,912 | 1,068 | 7,382,016 | 10 | 1/738 | 79 | 546,048 | 0 |  |
| ***HvKu70*** | 6,144 | 1,033 | 6,346,752 | 3 | 1/2116 | 2407 | 14,788,608 | 4 | 1/3697 |
| ***HvKu80*** | 5,376 | 913 | 4,908,288 | 12 | 1/409 | 307 | 1,650,432 | 0 |  |
| ***HvLSD1*** | 3,072 | 327 | 1,004,544 | 2 | 1/502 | 671 | 2,061,312 | 4 | 1/515 |
| ***HvPARP3*** | 5,376 | 1,272 | 6,838,272 | 4 | 1/1710 | 888 | 4,773,888 | 7 | 1/682 |
| ***HvPRT6*** | 6,912 | 711 | 4,914,432 | 8 | 1/614 | 372 | 2,571,264 | 3 | 1/857 |
| ***HvRAA1*** | 5,376 | 321 | 1,725,696 | 20 | 1/86 | 224 | 1,204,224 | 14 | 1/86 |
| ***HvRTH3*** | 6,912 | 962 | 6,649,344 | 5 | 1/1330 | 53 | 366,336 | 0 |  |
| ***HvSNAC1*** | 6,021 | 993 | 5,978,853 | 16 | 1/374 | 284 | 1,709,964 | 3 | 1/570 |
| ***HvUVRD*** | 6,144 | 450 | 2,764,800 | 4 | 1/691 | 427 | 2,623,488 | 5 | 1/525 |
| ***HvWRKY38*** | 3,072 | 600 | 1,843,200 | 4 | 1/461 | 134 | 411,648 | 2 | 1/206 |
| **Total** |  |  | 116,008,677 | 261 | **1/444** |  | 66,254,220 | 121 | **1/548** |
