# Supplementary material for: HorTILLUS—A Rich and Renewable Source of Induced Mutations for Forward/Reverse Genetics and Pre-breeding Programs in Barley (Hordeum vulgare L.)
Source: Front Plant Sci. 2018 Feb 21;9:216. doi: 10.3389/fpls.2018.00216 (PMC5826354; doi:10.3389/fpls.2018.00216)
Supplement: Supplementary file 4 [file Table4.DOCX]

Supplementary Table 4. TILLING populations developed for barley.

| **Cultivar/ line/ ecotype** | **Mutagen** | **Dose** | **Size of M_2_ population** | **Average No. of M_2_ plants analyzed** | **No. of genes analyzed** | **Total sequence screened (Mb)** | **Total No. of. mutations detected** | **Mutation density**  **(1 mutation per kb)** | **Mutation spectrum (%)** | | | | **Substitution types (%)** | | | **References** |
| --- | --- | --- | --- | --- | --- | --- | --- | --- | --- | --- | --- | --- | --- | --- | --- | --- |
|  |  |  |  |  |  |  |  |  | **Missense** | **Silent** | **Nonsense** | **Non –coding** | **Transitions G/C>A/T** | **Transitions**  **A/T>G/C** | **Transversions** |  |
| Sebastian | NaN_3_ + MNU | 1.5 mM NaN_3_/3h - 6h iig - 0.75 or 0.5 mM MNU/3h | 11,199 | 5123 | 32 | 182.2 | 382 | 477 | 41.9 | 25.6 | 0.8 | 31.7 | 88 | 4.5 | 7.5 |  |
| Optic | EMS | 20-30 mM/ 16 h | 9,216 | 9216 | 2 | 12.3 | 10 | 1,000 | 60 | 40 | 0 | 0 | 70 | 10 | 20 | Caldwell et al., 2004 |
| Barke | EMS | 20-60 mM/ 16 h | 10,279 | 7389 | 6 | 52.3 | 81 | 500* | 35.8 | 39.5 | 3.7# | 21 | n.a. | n.a. | n.a | Gottwald et al., 2009 |
| Morex | NaN_3_ | 10 mM/ 2 h | 4,906 | 3148 | 4 | 10.2 | 22 | 374* | 68.2 | 18.2 | 0 | 13.6 | 95.5 | 0 | 4.5 | Talame et al., 2008 |
| Morex | NaN_3_ | 10 mM/ 2 h | 4,906 | 4107 | 11 | 35.6 | 69 | 428* | 56.6 | 23.2 | 1.4 | 18.8 | 97.1 | 0 | 2.9 | Talame et al., 2009 |
| Lux | NaN_3_ | 2.5 mM/ 2.5 h | 9,575 | 9575 | 2 | 12.3 | 5 | 2,500 | 60 | 0 | 0 | 40 | 80 | 0 | 20 | Lababidi et al., 2009 |
| DH 930-36 | MNU | 0.5-1.5 mM/ 3 h | 1,372 | 1348 | 2 | 4.5 | 9 | 504 | 55.6 | 22.2 | 0 | 22.2 | 55.5 | 0 | 44.5 | Kurowska et al., 2012 |
| DH 930-36 | Gamma rays | 150-210 Gy | 1,753 | 1644 | 1 | 3.2 | 1 | 3,207 | - | 100 | 0 | 0 | 0 | 0 | 100 | Kurowska et al., 2012 |

*100-200 bp of each amplicon were excluded from the calculation of the total sequence length used for estimation of mutation density

n.a. – data not available

# nonsense and mutations in splice sites resulting in truncated protein
