# Supplementary figures and images for: HorTILLUS—A Rich and Renewable Source of Induced Mutations for Forward/Reverse Genetics and Pre-breeding Programs in Barley (Hordeum vulgare L.)
Source: Front Plant Sci. 2018 Feb 21;9:216. doi: 10.3389/fpls.2018.00216 (PMC5826354; doi:10.3389/fpls.2018.00216)

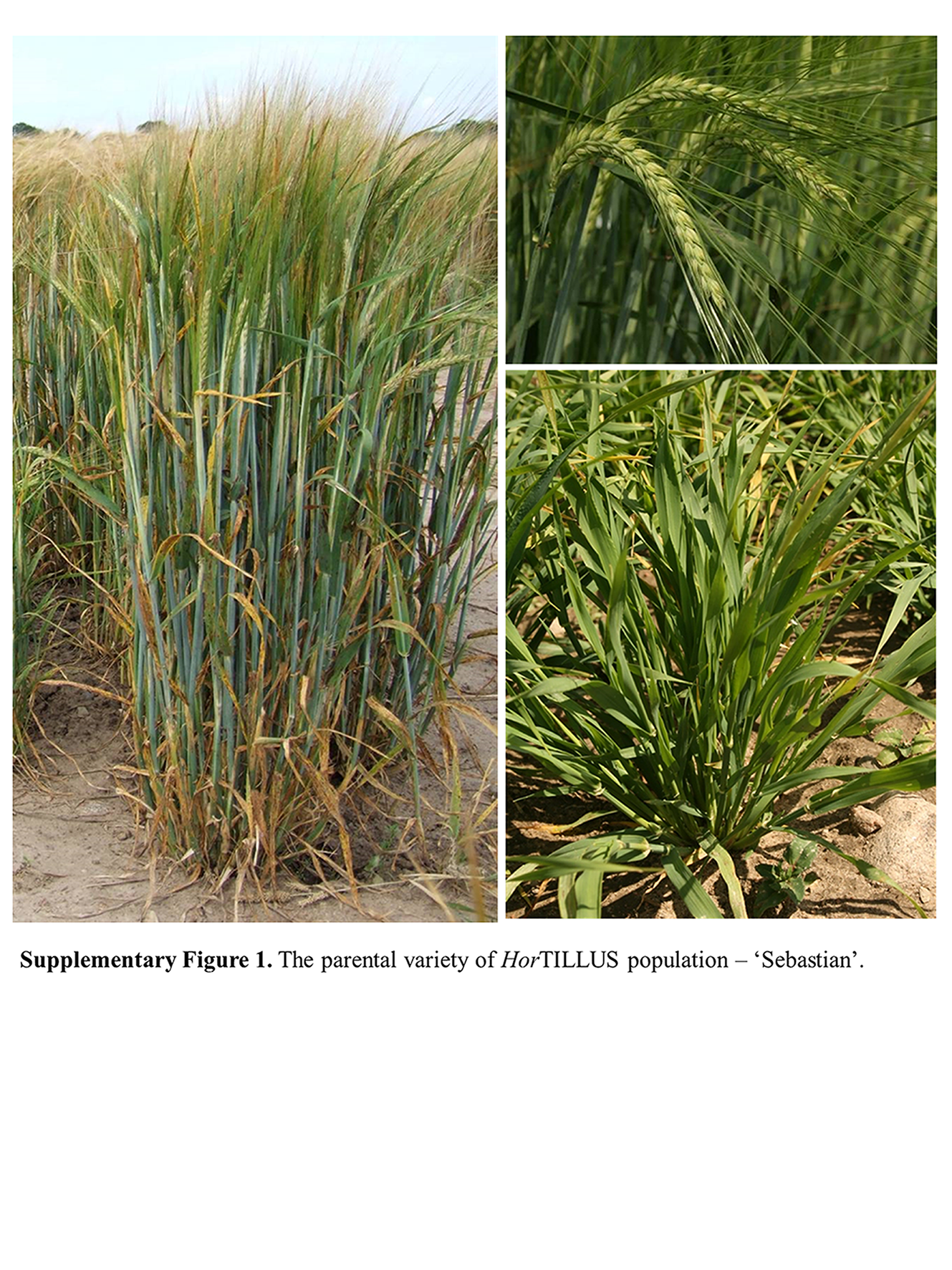

Supplement: Supplementary file 5 [file Image1.tif]

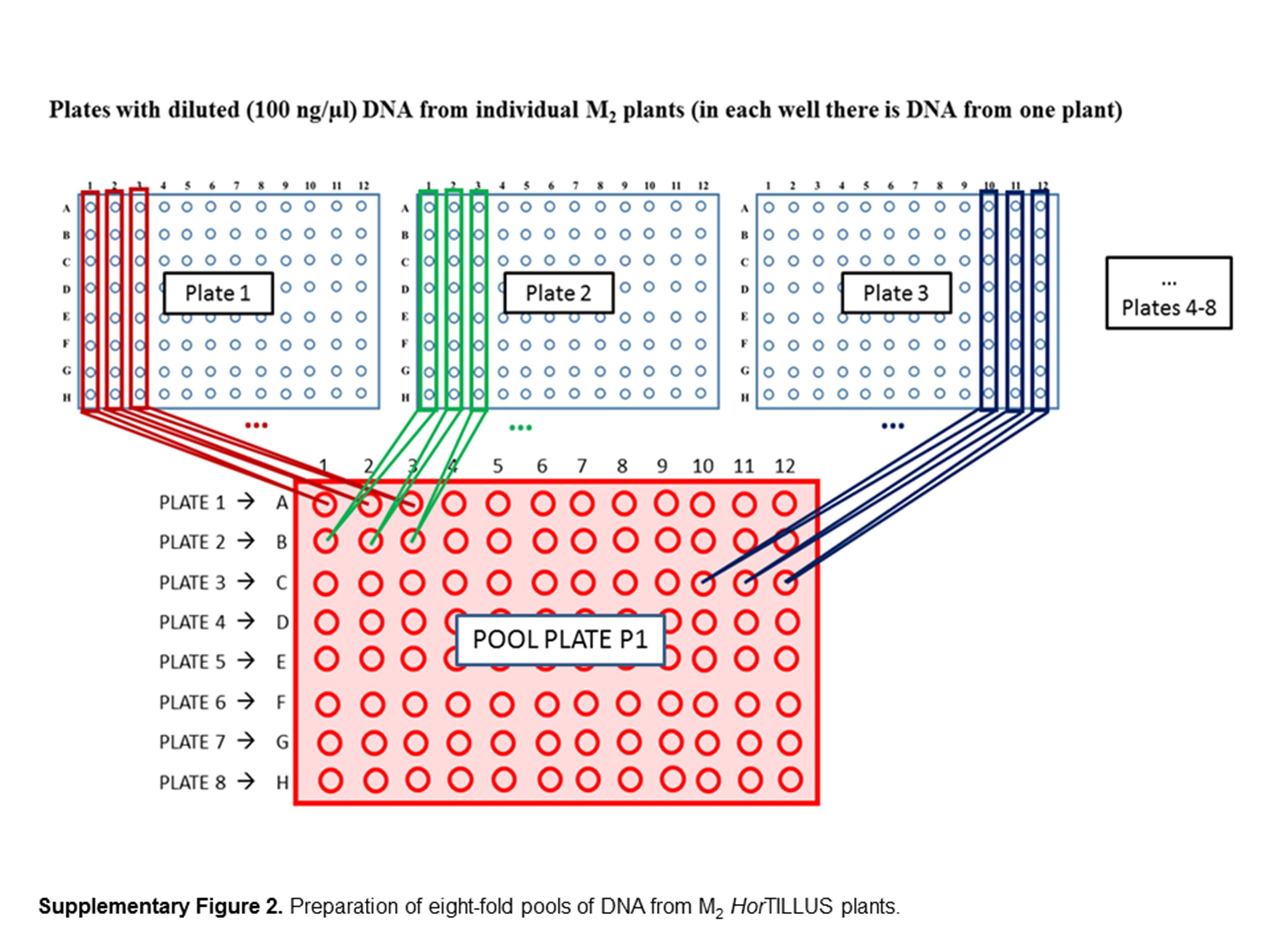

Supplement: Supplementary file 6 [file Image2.tif]

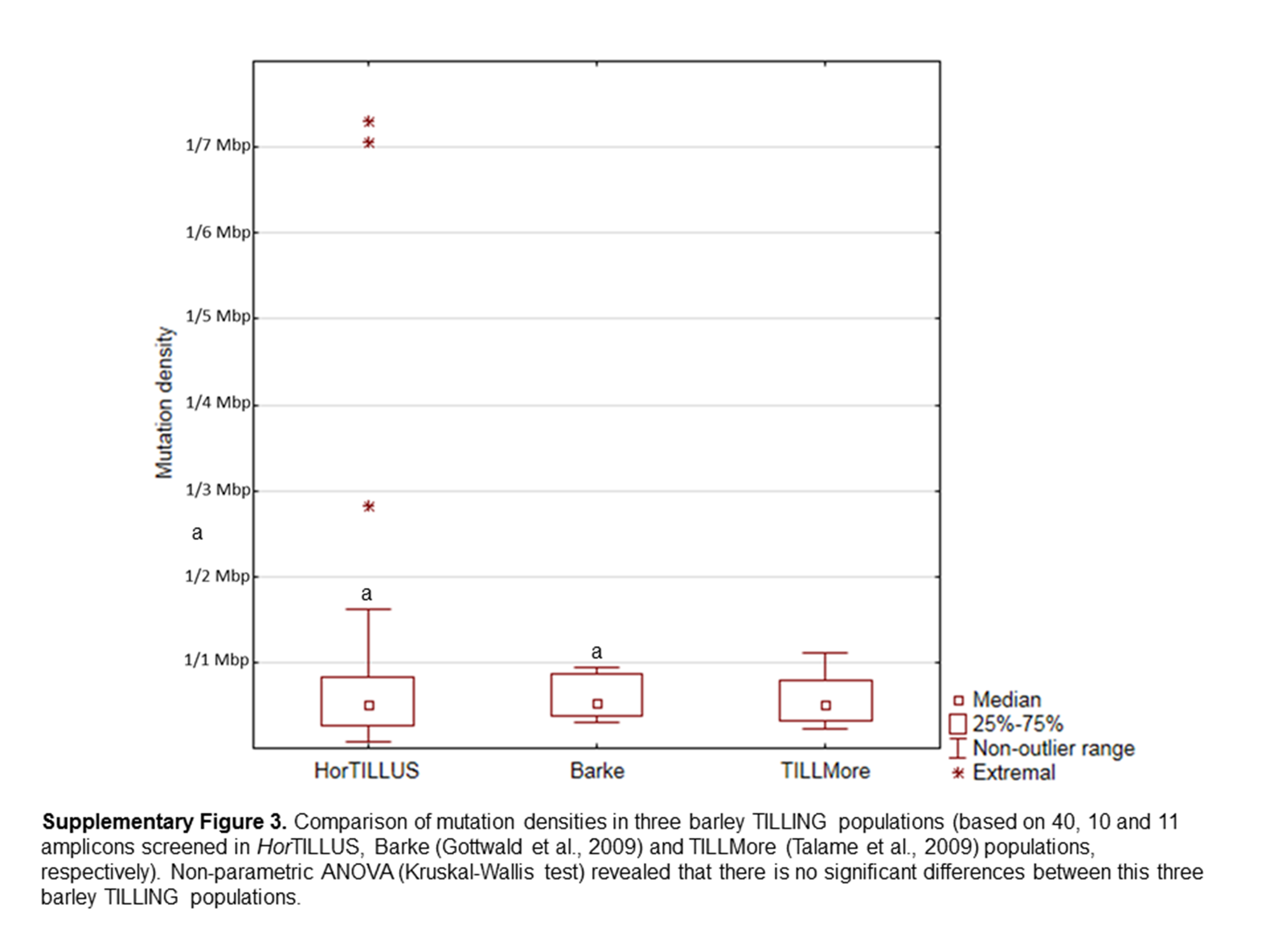

Supplement: Supplementary file 7 [file Image3.tif]
